# A New Dynamical Method for Bearing Fault Diagnosis Based on Optimal Regulation of Resonant Behaviors in a Fluctuating-Mass-Induced Linear Oscillator

**DOI:** 10.3390/s21030707

**Published:** 2021-01-21

**Authors:** Kehan Chen, Yuting Lu, Lifeng Lin, Huiqi Wang

**Affiliations:** 1 College of Mathematics and Statistics, Chongqing University, Chongqing 401331, China; kehanchen@cqu.edu.cn; 2 College of Big Data and Software Engineering, Chongqing University, Chongqing 401331, China; luyuting@cqu.edu.cn; 3 College of Computer and Information Science, Fujian Agriculture and Forestry University, Fuzhou 350002, China; linlf@fafu.edu.cn

**Keywords:** bearing fault diagnosis, linear oscillator (LO), generalized stochastic resonance (GSR), generalized scale transformation (GST), symmetric dichotomous noise (SDN)

## Abstract

Stochastic resonance (SR), a typical randomness-assisted signal processing method, has been extensively studied in bearing fault diagnosis to enhance the feature of periodic signal. In this study, we cast off the basic constraint of nonlinearity, extend it to a new type of generalized SR (GSR) in linear Langevin system, and propose the fluctuating-mass induced linear oscillator (FMLO). Then, by generalized scale transformation (GST), it is improved to be more suitable for exacting high-frequency fault features. Moreover, by analyzing the system stationary response, we find that the synergy of the linear system, internal random regulation and external excitement can conduct a rich variety of non-monotonic behaviors, such as bona-fide SR, conventional SR, GSR, and stochastic inhibition (SI). Based on the numerical implementation, it is found that these behaviors play an important role in adaptively optimizing system parameters to maximally improve the performance and identification ability of weak high-frequency signal in strong background noise. Finally, the experimental data are further performed to verify the effectiveness and superiority in comparison with traditional dynamical methods. The results show that the proposed GST-FMLO system performs the best in the bearing fault diagnoses of inner race, outer race and rolling element. Particularly, by amplifying the characteristic harmonics, the low harmonics become extremely weak compared to the characteristic. Additionally, the efficiency is increased by more than 5 times, which is significantly better than the nonlinear dynamical methods, and has the great potential for online fault diagnosis.

## 1. Introduction

Rolling bearing is one of the most critical aspects of modern machines, such as wind turbines, machine tools, centrifugal pumps, compressors, and motorized spindles [1]. In complex operating environments, the faults are inevitable and even lead to the damage of whole equipment. Thus, it is extremely important to monitor the health state and diagnose the early fault. In the past few decades, the issue of bearing fault diagnosis has attracted more and more attentions, and many methods are proposed in view of vibration, acoustics, liquid and deep learning [2,3,4,5]. Moreover, fault signals are always shown to be weak, especially in early stages. The ability to identify and extract weak signals become a key consideration. Some diagnosis methods focusing on noise suppression or cancellation technologies have been studied in depth and shown to be successful to highlight the weak fault features in many practical situations, i.e., maximum correlated kurtosis deconvolution [6], spectral kurtosis [7], empirical mode decomposition [8], wavelet transform [9], and chaos theory [10], but it is limited because of the inevitable damage of fault features during the de-noising process.

Recently, a new type of fault diagnosis methods based on non-monotonous dynamical behaviors are proposed in view of helpful randomness, such as stochastic resonance (SR) [11,12,13], vibration resonance [14,15], chaotic system [16,17], fractal method [18,19], etc. It is worth emphasizing that these dynamical methods have played an important role in modern engineering fields, and shown the intrinsic superiority in weak fault feature extraction by transferring the energy of noise to a weak signal [20,21]. Meanwhile, in order to overcome the limitation that classical resonant behaviors can only be applied to the low-frequency signal [22], some transformed systems have been considered, typically including scale transformation (ST) method [23,24], and generalized scale transformation (GST) [25,26,27], and they have been verified to be effective in the fault diagnosis of weak high-frequency signal. Recently, some adaptive algorithms, such as particle swarm optimization (PSO) and genetic algorithm (GA), are involved in the online conditions to optimize the system parameters based on the adaptive regulation of resonant behaviors [28,29].

However, all these dynamical methods mentioned above were based on nonlinear systems, in which nonlinearity, periodicity and random force were generally regarded as the basic elements for generating classical SR behaviors, but in recent years, many studies on wide-sense SR phenomena have overturned this view, and expanded it to linear oscillator (LO) by introducing additive and multiplicative noise, i.e., linear noisy oscillators with random fluctuations on damping [30,31], frequency [32,33,34], or mass [35,36]. Particularly, random-mass systems were first proposed in chemical and biological background, where the surrounding molecules not only collide with the oscillator but may also adhere to it, thereby changing its mass [37]. The additive noise and mass fluctuation are responsible for an influx of energy to the oscillator and its dissipation to the surrounding environment. It is found that the synergy could conduct a rich variety of generalized stochastic resonance (GSR) behaviors, played an important role in enhancing the driving signal in terms of output amplitude amplification (OAA) or signal-to-noise ratio (SNR). Henceforth, such a model has received widespread attention and shown prominent significance with the explosive development of micro- and nano-technologies, which leads to the growing need to study the influences of random mass disturbance on the systems, i.e., ion-ion reactions, electrodeposition, granular flow, film deposition, nano-technological devices, etc.

Therefore, we are inspired to consider a new type of GSR behavior based dynamical system for bearing fault diagnosis in the framework of linear oscillators, and extend it to the high-frequency signal by introducing GST method. Meanwhile, based on Shapiro-Loginov formula and Laplace transform, the system stationary response can be analyzed to obtain the exact expression of OAA. Moreover, an improved PSO algorithm [38] is adopted to adaptively achieve the optimal GSR match of system parameters, including damping coefficient, inherent frequency, the intensity and correlation rate of internal multiplicative fluctuation, and GST coefficient. Compared with the traditional methods, i.e., overdamped bistable SR system and underdamped Duffing oscillator in the simulations, the proposed method is verified to be significantly better in both output SNR performance with the known driving frequency and adaptive identifying ability with the unknown driving frequency. Finally, it also shows the superiority in the experimental application of bearing fault diagnosis. It is worth emphasizing that the proposed system can identify the characteristic more clearly in the most difficult diagnosis for rolling element fault, but the classical methods might be invalid. Meanwhile, the diagnosis efficiency is increased by more than 5 times, which is significantly better than the nonlinear dynamical methods. Obviously, it shows the great potential in engineering applications, especially the online fault diagnosis. The main novelty of this paper is to extend the classical SR based methods for bearing fault diagnosis to fluctuating-mass induced linear oscillator (FMLO), in which GST method is combined to compensate for the shortcomings of low-frequency driving constraint. It is verified from stationary response analysis that the proposed GST-FMLO system shows a rich variety of high-frequency induced GSR behaviors, particularly the double-peak bona fide SR with GST coefficient, which can be adaptively utilized in the multi-parameter regulation by PSO algorithm with the strategy of decreasing inertial weight, and achieve the optimal energy conversion from mass fluctuation to weak high-frequency fault characteristic.

The rest of this paper is organized as follows. In Section 2, we propose the system model, and reveal the GSR behaviors based on the analysis of system stationary response. In Section 3, the system implementation and optimal performance based on multiple-parameter regulation are investigated. In Section 4, the practical application and result discussions are presented. A brief conclusion follows in Section 5.

## 2. System Model

### 2.1. GST Based FMLO System

In some chemical and biological environments, it is a fact often observed that the surrounding molecules are capable of colliding with the Brownian particle and adhering to it randomly in the viscous medium, thereby forming the random fluctuation on particle mass [35,36,37]. Motivated by the phenomena, we consider the linear oscillator driven by a periodic signal u(t) and subjected to an additive noise ε(t) in the harmonic potential $U\left(x\right)=\frac{1}{2}\omega_{0}^{2}x^{2}$. Thus, the fluctuating-mass induced linear oscillator (FMLO) can be described by the following underdamped Langevin system:

(1) $$\left[1+\xi (t)\right]\ddot{x}\left(t\right)+\gamma_{0}\dot{x}\left(t\right)+\omega_{0}^{2}x\left(t\right)=g\left(t\right),$$

where x(t) is the system response at time *t*, $\gamma_{0}$ is a constant representing the damping coefficient, $\omega_{0}$ represents the system inherent frequency, g(t)=u(t)+ε(t) is the system input, including periodic signal u(t)=Acos(2πft) (with the amplitude *A* and driving frequency *f*) and the additive white Gaussian noise ε(t), which has the following statistical properties:

(2) 〈ε(t)〉=0,〈ε(t)ε(t+τ)〉=2Dδ(τ),

where *D* is the noise intensity. In addition, ξ(t) in Equation (1) represents the fluctuation on particle mass in Langevin dynamics, and it is modeled as symmetric dichotomous noise (SDN), taking two values $\xi \left(t\right)\;\in \;\left\{-\sigma_{0},\sigma_{0}\right\}$ with $\sigma_{0}\;\in \;\left(0,1\right)$ to ensure 1+ξ(t)>0. Meanwhile, it is supposed to satisfy

(3) $$\langle \xi \left(t\right)\rangle =0,\;\langle \xi \left(t\right)\xi \left(t+\tau \right)\rangle =\sigma_{0}^{2}e^{-\lambda_{0}\left|\tau \right|},$$

where $\sigma_{0}^{2}$ and $\lambda_{0}$ are the intensity and correlation rate of ξ(t), respectively. Because of different origins, multiplicative SDN fluctuation ξ(t) and additive noise ε(t) are further assumed to be independent, i.e., 〈ξ(t)ε(t+τ)〉=0.

Previous studies have noted that bona fide SR behavior widely exists in this type of LO system [31,32,34,36], that is, as the driving frequency *f* increases, the output periodic component of system stationary response non-monotonously decreases with single or double peaks, especially for high-frequency driving signal. It becomes much weaker, and completely submerges in the background noise. In the practical application of bearing diagnosis, the fault signals always behave to be high-frequency. Inevitably, in Equation (1) we need to apply the generalized scale transformation (GST), which has been verified to be effective in many dynamical methods for bearing fault diagnosis.

We introduce the coefficient *R* to perform time-scale transformation $\tilde{t}=Rt$, and define $y\left(\tilde{t}\right)\;≜\;x\left(t\right)$ in Equation (1), thus we have

(4) $$\left[1+\xi (\tilde{t})\right]\ddot{y}\left(\tilde{t}\right)+\frac{\gamma_{0}}{R}\dot{y}\left(\tilde{t}\right)+\frac{\omega_{0}^{2}}{R^{2}}y\left(\tilde{t}\right)=\frac{A}{R^{2}}\cos\left(2\pi \frac{f}{R}\tilde{t}\right)+\frac{1}{R^{2}}\varepsilon \left(\tilde{t}\right),$$

with the statistical properties: $\langle \xi \left(\tilde{t}\right)\rangle =0$, $\langle \varepsilon \left(\tilde{t}\right)\rangle =0$, $\langle \xi \left(\tilde{t}\right)\xi \left(\tilde{t}\;+\;\tilde{\tau }\right)\rangle =\sigma_{0}^{2}e^{-\frac{\lambda_{0}}{R}\left|\tilde{\tau }\right|}$, $\langle \varepsilon \left(\tilde{t}\right)\varepsilon \left(\tilde{t}\;+\;\tilde{\tau }\right)\rangle =2D\delta \left(\;\tilde{\tau }\right)$, and $\langle \xi \left(\tilde{t}\right)\varepsilon \left(\tilde{t}\;+\;\tilde{\tau }\right)\rangle =0$.

Comparing Equation (4) with Equation (1), we observe that the driving frequency is decreased to $\frac{f}{R}$, but the amplitude decreases to $\frac{A}{R^{2}}$ after the transformation. It is noted that the purpose of using re-scaled method is to find the suitable parameters to accomplish system resonance behaviors. As a result, to ensure that the transformed system has an equivalent dynamical behavior driven by the low-frequency signal, we have to make the input signal recover to the original strength [25,26,27]. Accordingly, the system described by Equation (1) is equivalently extended to

(5) $$\left[1+\tilde{\xi }\left(\tilde{t}\right)\right]\ddot{y}\left(\tilde{t}\right)+\gamma \dot{y}\left(\tilde{t}\right)+\omega^{2}y\left(\tilde{t}\right)=g\left(\tilde{t}\right),$$

where γ and ω are the regulatable parameters, which have the similar meanings as $\gamma_{0}$ and $\omega_{0}$ in Equation (1); $g(\tilde{t})$ is the system input, which includes the periodic signal $u\left(\tilde{t}\right)=A\cos\left(2\pi \tilde{f}\tilde{t}\right)$ (with lowered driving frequency $\tilde{f}=\frac{f}{R}$) and the background noise $\varepsilon (\tilde{t})$, satisfying the properties $\langle \varepsilon \left(\tilde{t}\right)\rangle =0$, $\langle \varepsilon \left(\tilde{t}\right)\varepsilon \left(\tilde{t}\;+\;\tilde{\tau }\right)\rangle =2D\delta \left(\;\tilde{\tau }\right)$); $\tilde{\xi }\left(\tilde{t}\right)$ is the internal random regulation of SDN on second order inertia term, and obeys: $\langle \tilde{\xi }\left(\tilde{t}\right)\varepsilon \left(\tilde{t}\;+\;\tilde{\tau }\right)\rangle =0$, $\langle \tilde{\xi }\left(\tilde{t}\right)\rangle =0$, and $\langle \tilde{\xi }\left(\tilde{t}\right)\tilde{\xi }\left(\tilde{t}\;+\;\tilde{\tau }\right)\rangle =\sigma^{2}e^{-\lambda |\tilde{\tau }|}$, in which $\sigma^{2}$ and λ are the intensity and correlation rate of $\tilde{\xi }\left(\tilde{t}\right)$.

Here Equation (5) is named GST-FMLO system. In practical applications, the measured high-frequency signal should be converted to low-frequency signal by selecting an appropriate GST coefficient *R*, and then it is processed by Equation (5) as the system input. Based on the wide-sense SR mechanism existed in LOs driven by additive and multiplicative noises, the input signal can be maximally amplified by optimizing the system parameters, i.e., γ, ω, σ, λ and *R*. In the following subsection, we will analyze the system stationary response in details to obtain the output amplitude amplification (OAA), and reveal the cooperative mechanism of multiple parameters induced generalized SR (GSR) behaviors in the proposed GST-FMLO system.

### 2.2. System Stationary Response

Firstly, we perform two operations upon Equation (5): (i) averaging Equation (5) with respect to the noise term $\varepsilon (\tilde{t})$, (ii) multiplying the both sides of Equation (5) by $\tilde{\xi }\left(\tilde{t}\right)$ and then averaging it. We obtain

(6) $$\begin{array}{c} \left\{\begin{array}{c} \frac{d^{2}}{d{\tilde{t}}^{2}}\langle y(\tilde{t})\rangle +\langle \tilde{\xi }\left(t\right)\ddot{y}\left(\tilde{t}\right)\rangle +\gamma \frac{d}{d\tilde{t}}\langle y(\tilde{t})\rangle +\omega^{2}\langle y(\tilde{t})\rangle =A\cos\left(2\pi \tilde{f}\tilde{t}\right),\\ \langle \tilde{\xi }\left(\tilde{t}\right)\ddot{y}\left(\tilde{t}\right)\rangle +\sigma^{2}\frac{d^{2}}{d{\tilde{t}}^{2}}\langle y(\tilde{t})\rangle +\gamma \langle \tilde{\xi }\left(\tilde{t}\right)\dot{y}\left(\tilde{t}\right)\rangle +\omega^{2}\langle \tilde{\xi }\left(\tilde{t}\right)y\left(\tilde{t}\right)\rangle =0. \end{array}\right. \end{array}$$

To perform the splitting of correlations in Equation (6), we employ the Shapiro-Loginov formula [39] with following forms:

(7) $$\frac{d}{d\tilde{t}}\langle \tilde{\xi }\left(\tilde{t}\right)y\left(\tilde{t}\right)\rangle =\langle \tilde{\xi }\left(\tilde{t}\right)\dot{y}\left(\tilde{t}\right)\rangle -\lambda \langle \tilde{\xi }\left(\tilde{t}\right)y\left(\tilde{t}\right)\rangle ,$$

and

(8) $$\frac{d}{d\tilde{t}}\langle \tilde{\xi }\left(\tilde{t}\right)\dot{y}\left(\tilde{t}\right)\rangle =\langle \tilde{\xi }\left(\tilde{t}\right)\ddot{y}\left(\tilde{t}\right)\rangle -\lambda \langle \tilde{\xi }\left(\tilde{t}\right)\dot{y}\left(\tilde{t}\right)\rangle ,$$

respectively. Combining Equations (6)–(8), and then taking the Laplace transform, we obtain

(9) $$\begin{array}{c} \left\{\begin{array}{c} \left(s^{2}+\gamma s+\omega^{2}\right)Y_{1}\left(s\right)+Y_{4}\left(s\right)=\frac{As}{s^{2}+4\pi^{2}{\tilde{f}}^{2}}+\left(s+\gamma \right)y_{1}\left(0\right)+{\dot{y}}_{1}\left(0\right),\\ \sigma^{2}s^{2}Y_{1}\left(s\right)+\omega^{2}Y_{2}\left(s\right)+\gamma Y_{3}\left(s\right)+Y_{4}\left(s\right)=s\sigma^{2}y_{1}\left(0\right)+\sigma^{2}{\dot{y}}_{1}\left(0\right),\\ sY_{2}\left(s\right)=Y_{3}\left(s\right)-\lambda Y_{2}\left(s\right)+y_{2}\left(0\right),\\ sY_{3}\left(s\right)=Y_{4}\left(s\right)-\lambda Y_{3}\left(s\right)+y_{3}\left(0\right), \end{array}\right. \end{array}$$

where $Y_{i}\left(s\right)=L\left\{y_{i}\left(\tilde{t}\right)\right\}\left(s\right)$, i=1,2,3,4, are the Laplace transforms of new variables: $y_{1}\left(\tilde{t}\right)\;≜\;\langle y(\tilde{t})\rangle$, $y_{2}\left(\tilde{t}\right)\;≜\;\langle \tilde{\xi }\left(\tilde{t}\right)y\left(\tilde{t}\right)\rangle$, $y_{3}\left(\tilde{t}\right)\;≜\;\langle \tilde{\xi }\left(\tilde{t}\right)\dot{y}\left(\tilde{t}\right)\rangle$, and $y_{4}\left(\tilde{t}\right)\;≜\;\langle \tilde{\xi }\left(\tilde{t}\right)\ddot{y}\left(\tilde{t}\right)\rangle$. $y_{1}\left(0\right)$, $y_{2}\left(0\right)$, $y_{3}\left(0\right)$ and ${\dot{y}}_{1}\left(0\right)$ are the initial conditions, whose influences will vanish in the long-time limit of $\tilde{t}\;\to \;\infty$. Thus, the solutions of Equation (9) can be easily obtained. Particulary, we have

(10) $$Y_{1}\left(s\right)=H\left(s\right)\frac{As}{s^{2}+4\pi^{2}{\tilde{f}}^{2}},$$

with

(11) $$\begin{array}{c} \left\{\begin{array}{c} H\left(s\right)=\frac{1}{D(s)}\left[{\left(s+\lambda \right)}^{2}+\gamma \left(s+\lambda \right)+\omega^{2}\right],\\ D\left(s\right)=\left(\omega^{2}+s^{2}+\gamma s\right)\left[{\left(s+\lambda \right)}^{2}+\gamma \left(s+\lambda \right)+\omega^{2}\right]-s^{2}\sigma^{2}{\left(s+\lambda \right)}^{2}. \end{array}\right. \end{array}$$

Based on linear response theory [40], we apply the inverse Laplace transform to obtain the asymptotic expression of system stationary response ${\langle y(\tilde{t})\rangle }_{\mathrm{as}}$ with the following form:

(12) $${\langle y(\tilde{t})\rangle }_{\mathrm{as}}=\langle y(\tilde{t})\rangle {|}_{\;\tilde{t}\to \infty }={\tilde{A}}_{\mathrm{as}}\cos\left(2\pi \tilde{f}\tilde{t}+{\tilde{\varphi }}_{\mathrm{as}}\right),$$

where ${\tilde{A}}_{\mathrm{as}}$, ${\tilde{\varphi }}_{\mathrm{as}}$ are the stationary amplitude and phase shift, respectively, and have been arranged as follows:

(13) $$\begin{array}{c} \left\{\begin{array}{c} {\tilde{A}}_{\mathrm{as}}=A\left|H\left(j2\pi \tilde{f}\right)\right|=A\sqrt{\frac{\mu_{1}^{2}+\mu_{2}^{2}}{\mu_{3}^{2}+\mu_{4}^{2}}},\\ {\tilde{\varphi }}_{\mathrm{as}}=\arctan\left(\frac{\mu_{2}\mu_{3}-\mu_{4}\mu_{1}}{\mu_{1}\mu_{3}+\mu_{2}\mu_{4}}\right), \end{array}\right. \end{array}$$

with the related coefficients:

(14) $$\begin{array}{cc} \; & \mu_{1}=-4\pi^{2}{\tilde{f}}^{2}+\lambda^{2}+\gamma \lambda +\omega^{2},\\ \; & \mu_{2}=2\pi \tilde{f}\left(2\lambda +\gamma \right),\\ \; & \mu_{3}=-\left(4\pi^{2}{\tilde{f}}^{2}-\omega^{2}\right)\left(\lambda^{2}+\gamma \lambda +\omega^{2}-4\pi^{2}{\tilde{f}}^{2}\right)-4\pi^{2}{\tilde{f}}^{2}\sigma^{2}\left(4\pi^{2}{\tilde{f}}^{2}-\lambda^{2}\right)-4\pi^{2}{\tilde{f}}^{2}\gamma \left(2\lambda +\gamma \right),\\ \; & \mu_{4}=16\pi^{3}{\tilde{f}}^{3}\lambda \sigma^{2}-2\pi \tilde{f}\left(4\pi^{2}{\tilde{f}}^{2}-\omega^{2}\right)\left(2\lambda +\gamma \right)+2\pi \tilde{f}\gamma \left(\lambda^{2}+\gamma \lambda +\omega^{2}-4\pi^{2}{\tilde{f}}^{2}\right). \end{array}$$

It is worth emphasizing that the result holds under the premise of σ∈(0,1), which causes no positive real part in the roots of D(s)=0. Thus, Equation (9) has stable solutions [40]. Here we further focus on the output amplitude amplification (OAA) to measure the magnification ability of weak high-frequency signal:

(15) $$G\left(\gamma ,\omega ,\sigma ,\lambda ,R\right)=\frac{1}{A}{\tilde{A}}_{\mathrm{as}},$$

which has been expressed as the function of regulatable parameters, γ, ω, σ, λ, and *R*.

### 2.3. Multi-Parameter Induced GSR Behaviors

The typical SR behavior reveals the effect of randomness on enhancing the response of nonlinear systems to weak periodic signal and making output SNR varying with the intensity be non-monotonic. But in recent studies, the term has been extended in the framework of linear systems [32,37]. To avoid the misunderstanding, generalized stochastic resonance (GSR) is used to focus on the non-monotonic dependence of system performances (i.e., stationary amplitude, OAA, output SNR) on various parameters.

**Figure 1:**
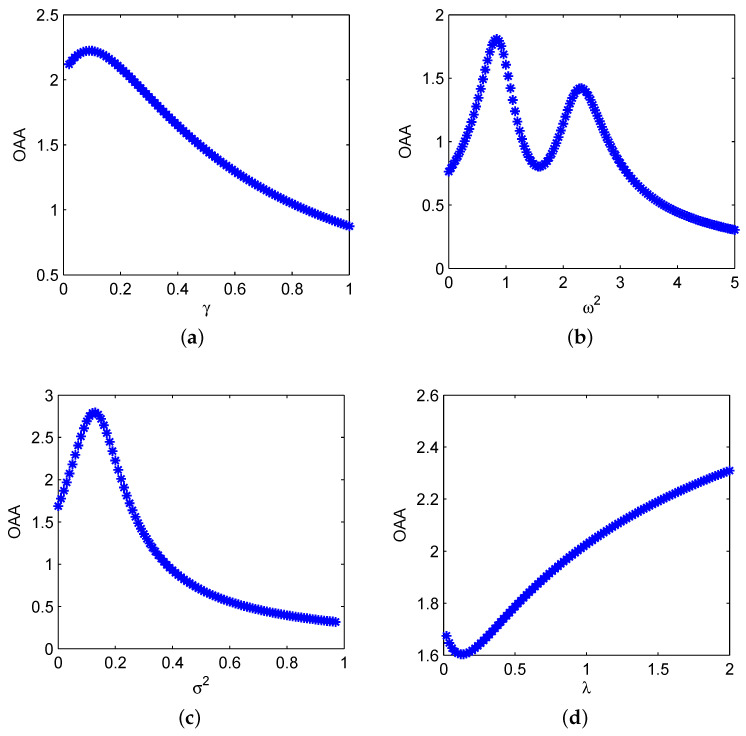
Dynamical behaviors of output amplitude amplification (OAA) varying with different system parameters: (**a**) *G* vs. γ with $\omega^{2}=1.0$, $\sigma^{2}=0.1$, λ=0.1, R=600, A=1.0, f=100, D=10; (**b**) *G* vs. $\omega^{2}$ with γ=0.2, $\sigma^{2}=0.2$, λ=0.1, R=500, A=1.0, f=100, D=10; (**c**) *G* vs. $\sigma^{2}$ with γ=0.1, $\omega^{2}=1.0$, λ=0.1, R=500, A=1.0, f=100, D=10; (**d**) *G* vs. λ with γ=0.1, $\omega^{2}=1.0$, $\sigma^{2}=0.2$, R=500, A=1.0, f=100, D=10.

In Figure 1, we plot the OAA curves varying with γ, $\omega^{2}$, $\sigma^{2}$, and λ, respectively. It is clearly observed that there exist multiple parameters induced SR or GSR behaviors, i.e., single-peak GSR of G(γ) in Figure 1a, double-peak GSR of $G(\omega^{2})$ in Figure 1b, classical SR of $G(\sigma^{2})$ in Figure 1c, SI of G(λ) in Figure 1d.

Specifically, in Figure 1a, we consider *G* varying with the damping coefficient γ, and observe the single-peak GSR behavior, based on which the value of γ can be regulated within [0.01,0.89] to magnify the stationary amplitude $A_{\mathrm{as}}$ by lowering the driving frequency to $\tilde{f}=\frac{f}{R}\;\approx \;0.1667$, and the optimum value occurs at γ=0.09, corresponding to the maximum G=2.224. In Figure 1b, *G* is plotted as the function of system inherent frequency $\omega^{2}$, and it typically shows double-peak GSR behavior. By confining $G(\omega^{2})\;>\;1$, we find that $\omega^{2}$ should be controlled into [0.28,1.28] or [1.92,2.76], and the optimum value of main-peak occurs at $\omega^{2}=0.84$ with the maximum G=1.809.

Furthermore, we depict the curves of *G* varying with the parameters of SDN fluctuation, i.e., $\sigma^{2}$ and λ, as shown in Figure 1c,d, respectively. Obviously, $G(\sigma^{2})$ behaves non-monotonous phenomenon of conventional SR, and the peak occurs at $\sigma^{2}=0.13$, with which the internal SDN energy can be optimally transformed to periodic signal at the lowered driving frequency $\tilde{f}=0.2$. It plays an important role in magnifying the signal, and it is similar to the additive noise in classical nonlinear bistable system [22]. Conversely, as λ increases in Figure 1d, we observe the non-monotonous SI phenomenon of first decrease and then increase, and the valley of maximum inhibition occurs at λ=0.12. Thus, it is necessary to avoid the area of SI valley in the parameter selection of λ.

**Figure 2:**
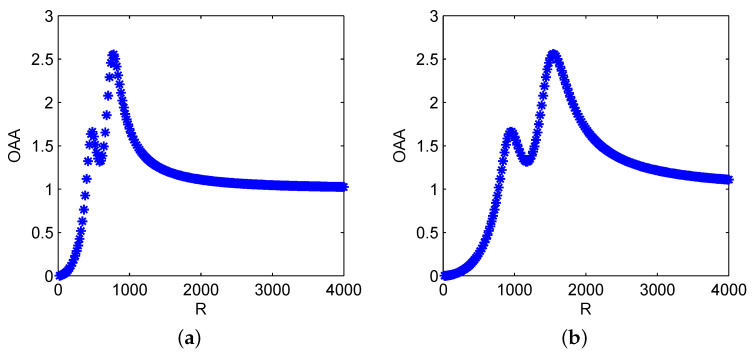
Dynamical behaviors of OAA varying with generalized scale transformation (GST) coefficient: (**a**) *G* vs. *R* with γ=0.2, $\omega^{2}=1.0$, $\sigma^{2}=0.2$, λ=0.1, A=1.0, f=100, D=10; (**b**) *G* vs. *R* with γ=0.2, $\omega^{2}=1.0$, $\sigma^{2}=0.2$, λ=0.1, A=1.0, f=100, D=10.

In Figure 2, we investigate the effect of GST coefficient *R* in the system driven by high-frequency signal, respectively with f=100 Hz and 200 Hz, and we find that as *R* increases, both the evolutions of G(R) show the bona-fide SR with double peaks. It can be explained as follows: in GST-FMLO system, the internal SDN fluctuation $\tilde{\xi }\left(\tilde{t}\right)\;\in \;\left\{-\sigma ,\sigma \right\}$ leads to two stable states in statistical sense, thus it can be regarded as an additive noisy oscillator [37], and approximately exists two resonance frequencies $f_{\mathrm{SR}}=\frac{R}{2\pi }\sqrt{\frac{\omega^{2}}{1\pm \sigma }\;-\;\frac{\gamma^{2}}{2{\left(1\pm \sigma \right)}^{2}}}$. As expected, in Figure 2a with f=100 Hz, we first observe the sub-peak at R=480, and the main-peak occurs at R=780, with which the signal can be optimally regulated to magnify 2.554 times in terms of OAA. In Figure 2b, where the driving frequency is increased to f=200 Hz and the other parameters remain the same as those in Figure 2a, it is seen that G(R) performs the similar non-monotonous tendency with the increase of *R*, and it is equivalent to the curve in Figure 2a stretched to 2 times in horizontal direction. Thus, the positions of two peaks proportionally shift to the larger value of *R*, i.e., R=960, and 1560, and the peak values remain unchanged. It shows that GST coefficient *R*, combined with γ, ω, σ and λ, can be regulated to match the external driving frequency *f*, and reach the main-peak of bona-fide SR to maximally enhance the input periodic signal.

## 3. Numerical Performance Based on Multi-Parameter Optimization

### 3.1. Numerical Implementation

Recalling Equation (5), the system can be rewritten as:

(16) $$\begin{array}{c} \left\{\begin{array}{c} z\left(\tilde{t}\right)=\dot{y}\left(\tilde{t}\right),\\ \left[1+\tilde{\xi }\left(\tilde{t}\right)\right]\dot{z}\left(\tilde{t}\right)+\gamma z\left(\tilde{t}\right)+\omega^{2}y\left(\tilde{t}\right)=g\left(\tilde{t}\right), \end{array}\right. \end{array}$$

which is the stochastic differential equations, and can be implemented by using fourth-order Runge-Kutta (RK-4) numerical method as follows:

(17) $$\begin{array}{c} \left\{\begin{array}{c} k_{1}=z\left[i\right],\;\;\;\;h_{1}=\frac{1}{1+\tilde{\xi }\left[i\right]}\left(-\gamma k_{1}-\omega^{2}y\left[i\right]+g\left[i\right]\right),\\ k_{2}=z\left[i\right]+\frac{1}{2}hh_{1},\;\;h_{2}=\frac{1}{1+\tilde{\xi }\left[i\right]}\left(-\gamma k_{2}-\omega^{2}\left(y\left[i\right]+\frac{1}{2}hk_{1}\right)+g\left[i\right]\right),\\ k_{3}=z\left[i\right]+\frac{1}{2}hh_{2},\;\;h_{3}=\frac{1}{1+\tilde{\xi }\left[i+1\right]}\left(-\gamma k_{3}-\omega^{2}\left(y\left(i\right)+\frac{1}{2}hk_{2}\right)+g\left[i\;+\;1\right]\right),\\ k_{4}=z\left[i\right]+hh_{3},\;\;\;h_{4}=\frac{1}{1+\tilde{\xi }\left[i+1\right]}\left(-\gamma k_{4}-\omega^{2}\left(y\left[i\right]+hk_{3}\right)+g\left[i\;+\;1\right]\right),\\ z\left[i\;+\;1\right]=z\left[i\right]+\frac{1}{6}h\left(h_{1}+2h_{2}+2h_{3}+h_{4}\right),\\ y\left[i\;+\;1\right]=y\left[i\right]+\frac{1}{6}h\left(k_{1}+2k_{2}+2k_{3}+k_{4}\right), \end{array}\right. \end{array}$$

where *h* is the iteration step, and y[i], z[i] and g[i] are the discrete forms of $y(\tilde{t})$, $z(\tilde{t})$ and $g(\tilde{t})$, respectively. Furthermore, the system performance of output SNR with the discrete form [12] is given by

(18) $$S\;N\;R_{\mathrm{out}}=10\log_{10}\frac{Y[\mathrm{round}(f/\Delta f)\;+\;1]}{\sum_{i=1}^{N_{s}/2}Y\left[i\right]-Y\left[\mathrm{round}\left(f/\Delta f\right)\;+\;1\right]},$$

where Y[i] is the amplitude spectrum calculated by fast Fourier transform (FFT) of discrete output y[i], Δf is the frequency resolution, and $N_{s}$ is the series length in FFT. Based on Equation (18), the input SNR, denoted as $S\;N\;R_{\mathrm{in}}$, can be similarly calculated by the FFT of g[i], and thus the SNR gain

(19) $$S\;N\;R_{\mathrm{gain}}=S\;N\;R_{\mathrm{out}}-S\;N\;R_{\mathrm{in}},$$

is used to measure the system performance on amplifying ability of weak high-frequency signal. If not specified in the simulations, the system is numerically achieved based on sampling frequency $f_{s}=$ 10,000 Hz and iteration step $h\;=R/f_{s}$, and the number of sampling points is set as $N_{s}=$ 100,000.

### 3.2. System Regulation Mechanism

Based on Equations (16)–(18), the numerical performance is explicitly or implicitly determined by different parameters, and thus the proposed GST-FMLO system can be optimized by the multi-parameter regulation, i.e., damping coefficient γ, inherent frequency ω, SDN parameters σ and λ, and GST coefficient *R*. In the following simulations, we mainly investigate the system regulation mechanisms on output SNR improvement. The input periodic signal is characterized by A=0.1, f=100 Hz, and submerged in the background noise with D=1.0.

- Damping Regulation

**Figure 3:**
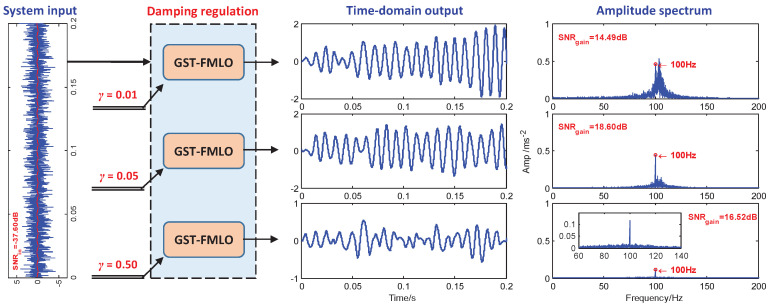
The performance of the generalized scale transformation-fluctuating-mass induced linear oscillator (GST-FMLO) system regulated by different damping coefficients γ=0.01, 0.05 and 0.50. The other parameters are chosen as ω=1.6, σ=0.2, λ=1.0, R=400, A=0.1, f=100, D=1.0.

In Langevin dynamics, the damping coefficient as an important physical parameter characterizes the energy dissipation, thus the small γ has the obvious magnification effect on the system input, for both the periodic signal and background noise. With the cooperation of internal SDN regulation, the system shows two equilibria in statistical sense, and a part of additive noise energy can be transformed into signal energy, which might lead to the increase of output SNR, i.e., $S\;N\;R_{\mathrm{gain}}=18.60$ dB for γ=0.05 in Figure 3. Although the output is relatively inhibited to a certain extend (compared with γ=0.01), it is also clearly observed the noise interference is decreased to replenish the inhibited signal energy. This is why we observe that the component is substantially retained at f=100 Hz.

However, much smaller γ, i.e., γ=0.01 in Figure 3, will over-amplify the noise, and the residual noise is still heavy and will submerge the periodic signal without the cooperation of other regulatable means. Thus, we observe the performance degradation $S\;N\;R_{\mathrm{gain}}=14.49$ dB instead. In contrast, with the increase of γ, i.e., γ=0.50, the system shows a significant impact on inhibiting the input, in which the inhibited periodic signal is too weak to excite the system to escape the local equilibria and regularly switch between two equilibria, even with the cooperation of input noise, which is still inhibited by the system at the same time. Thus, this also leads to a 2.08 dB decrease of SNR gain.

- Inherent Frequency Regulation

**Figure 4:**
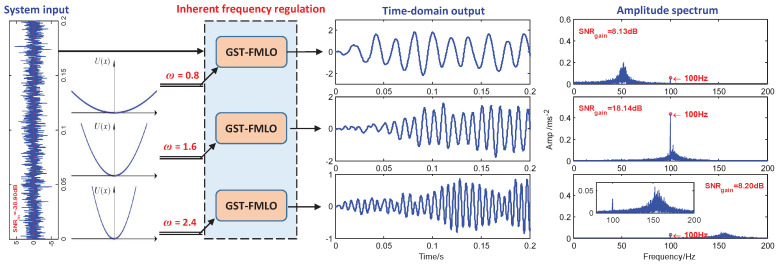
The performance of GST-FMLO system regulated by different inherent frequencies ω=0.8, 1.6 and 2.4. The other parameters are chosen as γ=0.05, σ=0.2, λ=1.0, R=400, A=0.1, f=100, D=1.0.

In Figure 4, we investigate the effect of potential $U\left(x\right)=\frac{1}{2}\omega^{2}x^{2}$, respectively regulated by inherent frequencies ω=0.8, 1.6 and 2.4. Here it is worth noting that U(x) is different from the potentials in classical bistable SR system [22], and nonlinear monostable SR system [41]. As a generalized monostable form, the SR behavior does not exist without the cooperation of multiplicative SDN. Thus, it is not involved in the conventional SR based bearing fault diagnosis methods. It is seen from Figure 4 that ω plays a role of band pass filter parameter in detecting the weak signal. As ω increases from 0.8 to 2.4, the pass band gradually shifts from lower band [30,70] to higher [130,170]. The input signal can be amplified significantly if ω matches with the driving frequency f=100 Hz, that is, it exactly falls into the regulated pass band [80,120] with ω=1.6.

- SDN Regulation

**Figure 5:**
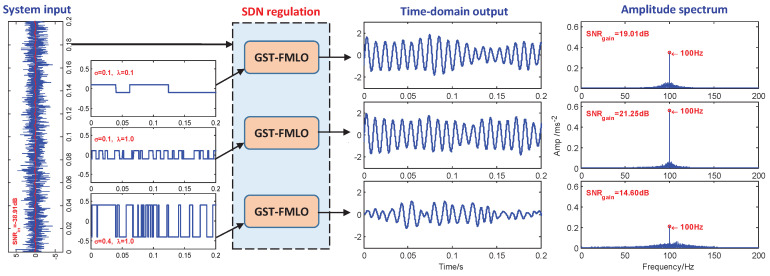
The performance of GST-FMLO system regulated by different SDN with parameters (σ,λ)=(0.1,0.1), (0.1,1.0) and (0.4,1.0). The other parameters are chosen as γ=0.1, ω=1.6, R=400, A=0.1, f=100, D=1.0.

Moreover, in Figure 5 we discuss the regulation effect of SDN fluctuation, which is randomly governed by fluctuating amplitude σ and correlation rate λ. When SDN is controlled by σ=0.1, then λ=0.1, which corresponds to the lower switching frequency in statistical sense, and it is difficult to transform noise energy to the relative high-frequency signal (with $\tilde{f}=\frac{f}{R}=0.25$ Hz). As λ increases to 1.0, we clearly observe the component at f=100 Hz is enhanced, and SNR gain is improved from 19.01 dB to 21.25 dB. On the other hand, if we further regulate SDN intensity, increased to $\sigma^{2}=0.4^{2}$, the residual noise energy plays a negative role in disordering the system response, thus we observe the component at f=100 Hz weakens, and the interference spreads to wider frequency band, which instead makes the SNR gain decrease to 14.60 dB.

- GST Regulation

**Figure 6:**
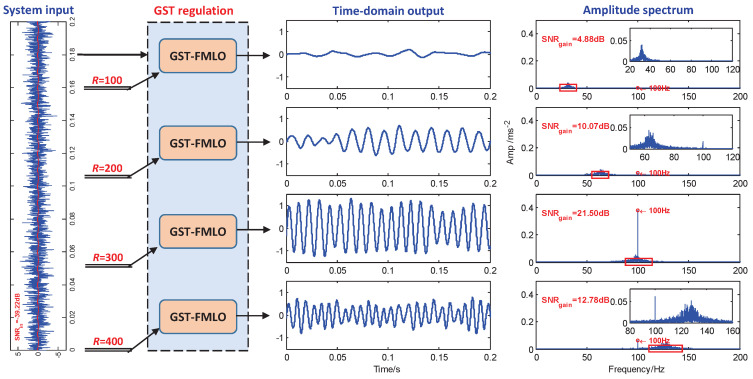
The performance of the GST-FMLO system regulated by different GST coefficients R=100, 200, 300 and 400. The other parameters are chosen as γ=0.1, ω=2.0, σ=0.1, λ=1.0, A=0.1, f=100, D=1.0.

Finally, we further analyze the regulation effect of GST coefficient *R* in Figure 6. In FMLO system, ω mainly controls the system to match with external driving frequency *f*, and leads to the amplification of weak signal based on bona fide SR behavior existing in the system. However, the effect is limited, that is, as ω increases, the output periodic component non-monotonously fades with double-peaks, especially for high-frequency driving signal, when it becomes much weaker, and even completely submerges in the background noise. Thus, GST is necessary, and *R* can be used to lower the driving frequency to arbitrarily expected value. In Figure 6, the system is regulated by *R*, respectively with R=100, 200, 300 and 400. As expected, we observe the similar regulation effect of band pass filter, and they respectively lead to the different pass bands from low to high. When the driving frequency f=100 Hz happens to fall into the band, i.e., [90,115] with R=300, we observe that noise energy out of the band can be effectively transformed into the energy of the periodic driving signal. Accordingly, the SNR gain is improved to 21.50 dB.

### 3.3. PSO Based Multi-Parameter Regulation

Based on the above description and discussion, we have different means and methods to improve the system performance for detecting the weak high-frequency signal (at the driving frequency $f=f_{\mathrm{in}}$) in the background noise. Taken together, all the parameters involved in Section 3.2 can be optimized based on $S\;N\;R_{\mathrm{out}}$, and the objective function is expressed as:

(20) $${\left\{\gamma ,\omega ,\sigma ,\lambda ,R\right\}}_{\mathrm{opt}}^{\mathrm{GST}-\mathrm{FMLO}}=\arg\max_{\gamma ,\omega ,\sigma ,\lambda ,R\in R^{+}}S\;N\;R_{\mathrm{out}}\left(\gamma ,\omega ,\sigma ,\lambda ,R\;|\;f=f_{\mathrm{in}}\right).$$

**Figure 7:**
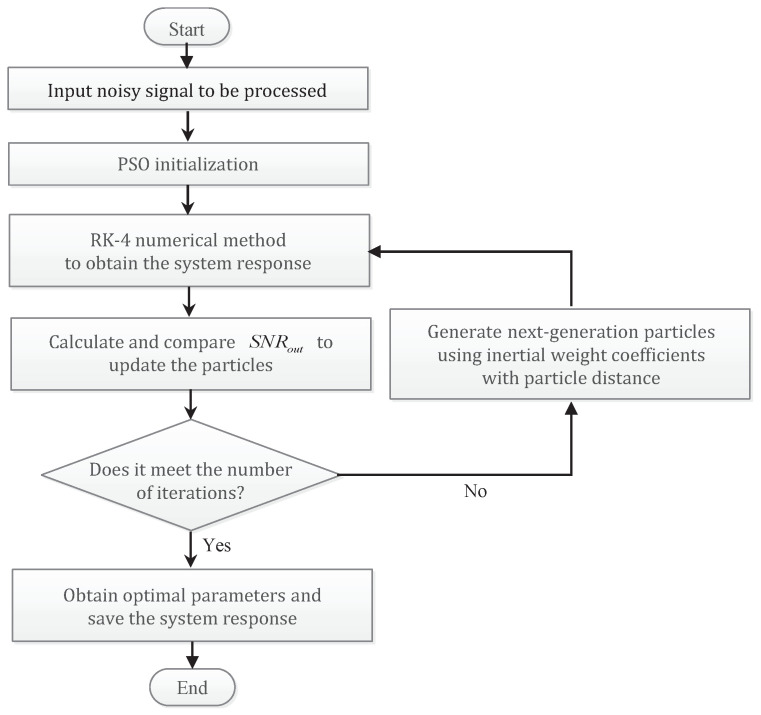
The skeleton diagram of multi-parameter optimization based on improved particle swarm optimization (PSO) algorithm.

By applying PSO algorithm [38], it is easy to obtain the optimal parameters. Moreover, the strategy of decreasing inertial weight based on particle distance is used to improve the global search capabilities, and avoid trapping in the local optimum, i.e., the value of ω or *R* at the sub-peak position, as shown in Figure 1b and Figure 2. The skeleton diagram is shown in Figure 7.

In order to verify the effectiveness of proposed method, two more classical SR systems are considered to compare the performance in the detection of weak high-frequency signal:

- overdamped bistable SR system (GST-OBSR)
  (21) $$\gamma \dot{y}\left(\tilde{t}\right)-ay\left(\tilde{t}\right)+by^{3}\left(\tilde{t}\right)=g\left(\tilde{t}\right),$$
  with four parameters: γ, *a*, *b*, *R*;
- underdamped Duffing oscillator (GST-Duffing)
  (22) $$\ddot{y}\left(\tilde{t}\right)+\gamma \dot{y}\left(\tilde{t}\right)-ay\left(\tilde{t}\right)+by^{3}\left(\tilde{t}\right)=g\left(\tilde{t}\right),$$
  with four parameters: γ, *a*, *b*, *R*;
- our proposed GST-FMLO system Equation (5) with five parameters: γ, ω, σ, λ, *R*.
  It is noted that, all the parameters in GST-OBSR and GST-Duffing systems are also optimized by the previously mentioned PSO algorithm with the objective function $S\;N\;R_{\mathrm{out}}$. In practical applications, the actual input driving frequency may be known, or unknown and should be estimated. Thus, in the following simulations, we focus on the performance of two cases, respectively with known and unknown driving frequency.

- Performance with Known Driving Frequency

**Figure 8:**
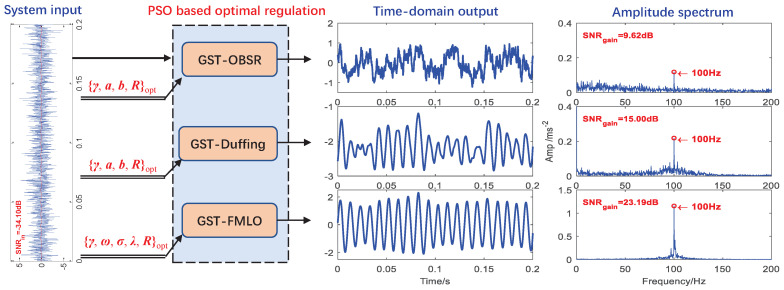
The optimal performance of three different dynamical methods (GST-overdamped bistable SR system (OBSR), GST-Duffing and GST-FMLO) in the detection of weak signal with the known driving frequency f=100 Hz.

We consider the optimal detection of weak signal in the background noise with D=1.0. In Figure 8, the noisy signal as the input is respectively processed by above three different systems, and the input SNR is calculated as −34.10 dB. By maximizing the $S\;N\;R_{\mathrm{out}}$ at the known driving frequency f=100 Hz in PSO algorithm, the parameters of three systems can be optimized, and the results are summarized in Table 1. With the optimal parameters, the system output and corresponding amplitude spectrum have been depicted in Figure 8. Obviously, compared with the results in Section 3.2, the weak signal has been further enhanced through the optimal GST-FMLO system with multi-parameter regulation, and the output SNR is improved to −10.91 dB with SNR gain 23.19 dB. Moreover, the other two systems is relatively difficult to deal with low harmonics, resulting in lower output SNR. Surprisingly, the linear system GST-FMLO can greatly amplify the characteristic harmonic, which causes the low harmonics to be extremely weak compared to the characteristic. This is quite valuable in bearing fault diagnosis.

- Adaptive Performance with Unknown Driving Frequency

In practical applications on bearing fault diagnosis, due to the inevitable influence of loads, sensor error and installation position, the deviation always exists between the theoretical and actual values of fault frequency. Thus, we need to adaptively estimate the actual fault frequency. Based on PSO algorithm in GST-FMLO system, the estimate is achieved by

(23) $$\begin{array}{c} \left\{\begin{array}{c} \hat{f}=\arg\max_{f\in [f_{a},f_{b}]}S\;N\;R_{\mathrm{out}}^{\mathrm{opt}}\left(f\right),\\ S\;N\;R_{\mathrm{out}}^{\mathrm{opt}}\left(f\right)=S\;N\;R_{\mathrm{out}}\left(f\in \left[f_{a},f_{b}\right]\;|\;{\left\{\gamma ,\omega ,\sigma ,\lambda ,R\right\}}_{\mathrm{opt}}^{\mathrm{GST}-\mathrm{FMLO}}\right). \end{array}\right. \end{array}$$

Obviously, the identifying ability of actual driving frequency is an important performance index, and it can be measured by analyzing whether the value of $S\;N\;R_{\mathrm{out}}^{\mathrm{opt}}$ at the actual value $\hat{f}=f_{\mathrm{in}}$ is significantly larger than $S\;N\;R_{\mathrm{out}}^{\mathrm{opt}}\left(\hat{f}\;\in \;\left[f_{a},f_{b}\right]\setminus f_{\mathrm{in}}\right)$.

**Figure 9:**
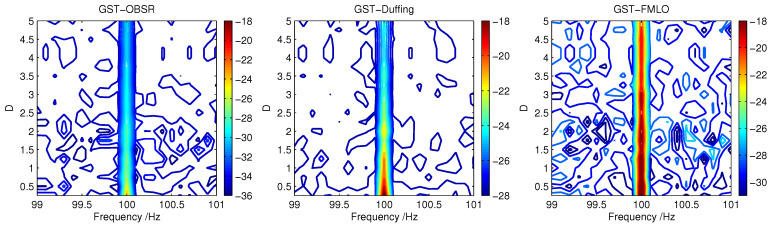
The adaptive performance of three different dynamical methods (GST-OBSR, GST-Duffing and GST-FMLO) in the detection of weak signal with the unknown driving frequency f=100 Hz.

We consider the adaptive performance of three different dynamical methods, i.e., GST-OBSR, GST-Duffing and GST-FMLO, in the detection of weak signal with the actual driving frequency $f_{\mathrm{in}}=100$ Hz, which is regarded as an unknown parameter. As the background noise intensity *D* varies within the range of [0.25,5.0], we observe the result of $S\;N\;R_{\mathrm{out}}^{\mathrm{opt}}\left(\hat{f}\;\in \;\left[99,101\right]\right)$, and depict $S\;N\;R_{\mathrm{out}}^{\mathrm{opt}}$ in $\hat{f}$-*D* plane in Figure 9, where the value can be determined by the color bar at the right side of each sub-figure. By comparing the color variation, the peak of $S\;N\;R_{\mathrm{out}}^{\mathrm{opt}}$ can be roughly identified at $f_{\mathrm{in}}=100$ Hz for the relative lower noise intensities. In the GST-OBSR and GST-Duffing systems, the peak value decreases significantly with the increase of *D*, and it becomes difficult to be clearly identified and accurately estimated. However in the GST-FMLO system, the decrease is limited as *D* increases, and with the increase of deviation $\delta_{f}=\left|\hat{f}\;-\;f_{\mathrm{in}}\right|$, $S\;N\;R_{\mathrm{out}}^{\mathrm{opt}}$ fades quickly. Thus, we can evidently distinguish the peak at the actual driving frequency $f_{\mathrm{in}}=100$ Hz, even under the background of much heavier noise.

It should be stated that the simply noisy sinusoids are considered in these simulations for convenience. The practical bearing fault signals are more complicated, as they are always non-stationary or cyclo-stationary [42]. Hence some preprocessing methods should be applied to make the system be more effective in the applications, which will be further described and discussed in next section.

## 4. Experimental Applications

**Figure 10:**
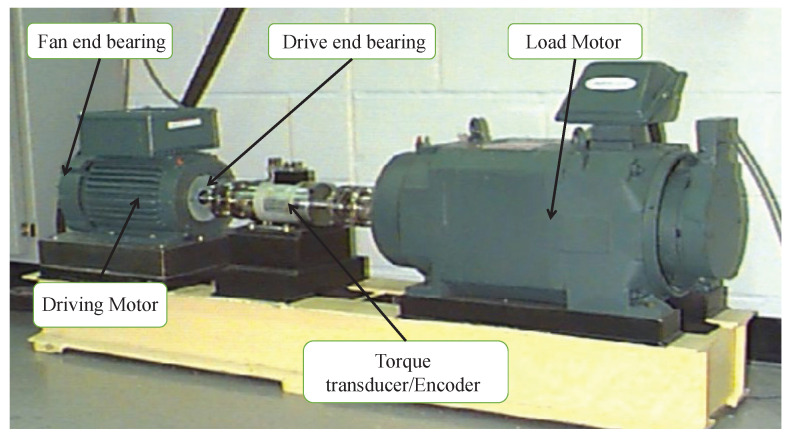
The basic layout of experimental setup.

In this section, we introduce the adaptive method of GST-FMLO system in bearing fault diagnosis, and verify the effectiveness and practicability. The experimental data comes from the tests conducted by Bearing Data Center of Case Western Reserve University [43], and the basic layout of experimental setup is shown in Figure 10. It consists of a 2 hp motor (left) driving the shaft where a torque transducer/encoder (center) are mounted. Torque is applied to the shaft via a dynamometer (right) and electronic control system. By using electro-discharge machining (EDM), the faults were seeded on the drive end bearing (SKF 6205-2RS JEM: inner ring diameter 0.9843 inches, outer ring diameter 2.0472 inches, rolling element diameter 0.3126 inches, pitch diameter 1.5370 inches, contact angle $0^{\circ }$, number of rolling elements 9), which leads to the fault size 0.021 inches at inner race, 0.007 inches at outer race, and 0.028 inches at rolling element. The faulted bearings were reinstalled into the test rig, which was run at the speed of 1797 rpm (i.e., $f_{r}=29.95$ Hz). When the bearing fault appears, periodic impulses can be revealed in the corresponding spectrum of the generated vibrational or acoustic signals, collected by using a 16 channel DAT recorder with sampling frequency $f_{s}=$ 12,000 Hz and sampling number $N_{s}=$ 120,000. Based on theoretical calculation in normal conditions without considering the influence of loads [42], the values of fault frequencies from inner race, outer race, and rolling element, are expected at $f_{\mathrm{BPFI}}=162.2$ Hz, $f_{\mathrm{BPFO}}=107.4$ Hz and $2f_{\mathrm{BSF}}=141.2$ Hz, respectively.

**Figure 11:**
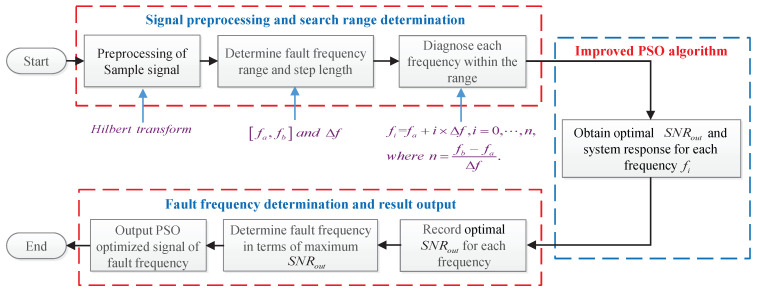
The skeleton diagram of numerical implementation for adaptive bearing fault diagnosis based on the improved PSO algorithm.

When a localized fault appears in a bearing with constant shaft speed, the collisions between the kinetic bearing components will generate a series of periodic or qusi-periodic impacts, which can be captured by the accelerometer or microphone sensors that are placed on or near to the bearing housing, but are always blurred by background noises [12]. Hence, the practical bearing fault signals satisfy the input requirement of SR or GSR based dynamical systems, i.e., GST-OBSR, GST-Duffing, GST-FMLO. Given this, the above three methods are suitable for processing the noisy bearing fault signals, and extracting the fault features from original signals, or envelope signals of the bearings [42]. Additionally, in terms of specific situation, the actual measured fault frequencies are distinguished from the theoretical values. They are should be identified adaptively in practice, and the skeleton diagram for adaptive bearing fault diagnosis has been described in Figure 11, where the difference between the applications of three systems is just the RK-4 numerical method to solve system response in the PSO algorithm.

**Figure 12:**
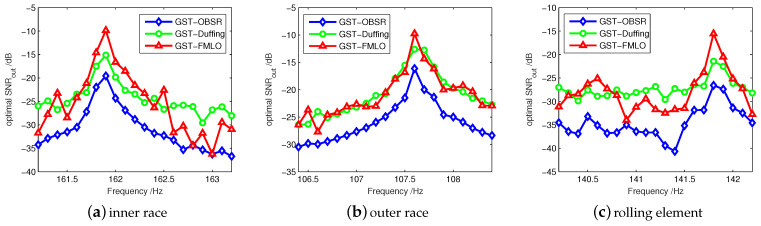
The adaptive identification of bearing fault frequencies ${\hat{f}}_{\mathrm{BPFI}}$, ${\hat{f}}_{\mathrm{BPFO}}$ and $2{\hat{f}}_{\mathrm{BSF}}$ in terms of optimal $S\;N\;R_{\mathrm{out}}$.

Based on the SR or GSR mechanism, the periodic signal can be maximally enhanced at the actual driving frequency in the systems, respectively with optimal parameters. Thus, we focus on the adaptively ability to identify bearing fault frequency. In Figure 12a–c, we respectively observe the optimal output SNR at a certain range around theoretical values, and plot the curves of $S\;N\;R_{\mathrm{out}}^{\mathrm{opt}}$ to estimate the actual fault frequencies based on the peaks. It is found that the actual fault frequency from inner race is ${\hat{f}}_{\mathrm{BPFI}}=161.9$ Hz, which is smaller than the theoretical value. However, the actual fault frequencies are higher than the theoretical results for outer race and rolling element, i.e., ${\hat{f}}_{\mathrm{BPFO}}=107.6$ Hz, $2{\hat{f}}_{\mathrm{BSF}}=141.8$ Hz. Moreover, the identifying ability of three different systems is basically consistent with the results in Figure 9. In all cases, the proposed GST-FMLO system shows the optimal identifying performance with the most distinctive peaks.

**Figure 13:**
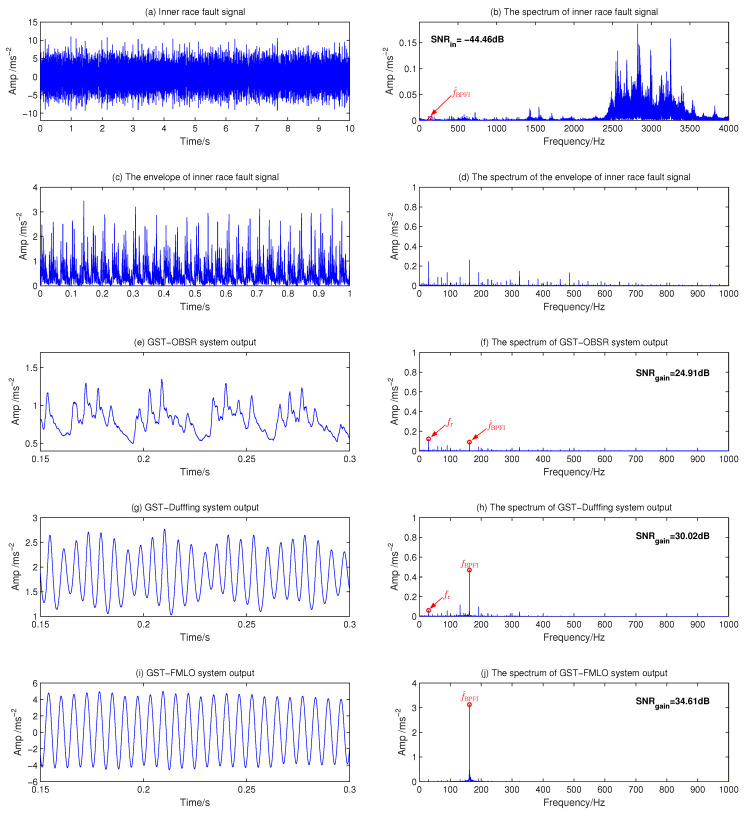
Inner race fault diagnosis by three different dynamical methods: (**a**) Inner race fault signal with ${\hat{f}}_{\mathrm{BPFI}}=161.9$ Hz; (**b**) The spectrum of inner race fault signal; (**c**) The envelope of inner race fault signal; (**d**) The spectrum of envelope signal; (**e**) GST-OBSR system output; (**f**) The spectrum of GST-OBSR system output; (**g**) GST-Duffing system output; (**h**) The spectrum of GST-Duffing system output; (**i**) GST-FMLO system output; (**j**) The spectrum of GST-FMLO system output.

Firstly, the vibration signal of a bearing with fault on the inner race is employed to compare the diagnosis performance of three different dynamical systems. The original waveform of the inner race fault signal and amplitude spectrum have been described in Figure 13a,b, respectively. It is seen that the noise influence is obvious in both time-domain and spectrum diagram, where the input SNR is −44.46 dB by numerical calculation, and the component at ${\hat{f}}_{\mathrm{BPFI}}=161.9$ Hz is extremely insignificant, which inevitably leads to much difficulty for recognizing the fault feature. As the preprocessing method, we analyze the envelope signal in Figure 13c,d, and the characteristic frequency is still difficult to identify due to excessive interference harmonics. It is reflected in the time-domain waveform that the noise covers the periodic component of characteristic frequency. In Figure 13e–h, we consider two traditional methods, that is, make the fault signal as input drive the GST-OBSR and GST-Duffing systems. It is intuitively seen from Figure 13e–h that, although the interferences are suppressed to a certain extent, some low harmonics (i.e., $f_{r}$ etc.) are still quite obvious, which seriously affects the identification of characteristic harmonics. Among them, due to the second order filtering effect of GST-duffing, the result will be slightly better [24]. It is also supported by the numerical results of output SNR, which is respectively improved to −19.55 dB and −14.44 dB, with the gains 24.91 dB and 30.02 dB. In Figure 13i,j, we investigate the diagnosis performance of the GST-FMLO system. Obviously, the component absolutely predominates at the fault frequency ${\hat{f}}_{\mathrm{BPFI}}=161.9$ Hz, and the low harmonics are not significantly observed. This is because of the co-excitation of internal and external noise energy, the linear system GST-FMLO greatly amplifies the characteristic harmonics, thus showing that the low-order harmonics become extremely weak relative to the characteristic. Based on this method, the output SNR can be further improved to −9.85 dB with SNR gain 34.61 dB, which demonstrates that GST-FMLO system performs better in processing the inner race fault signal.

**Figure 14:**
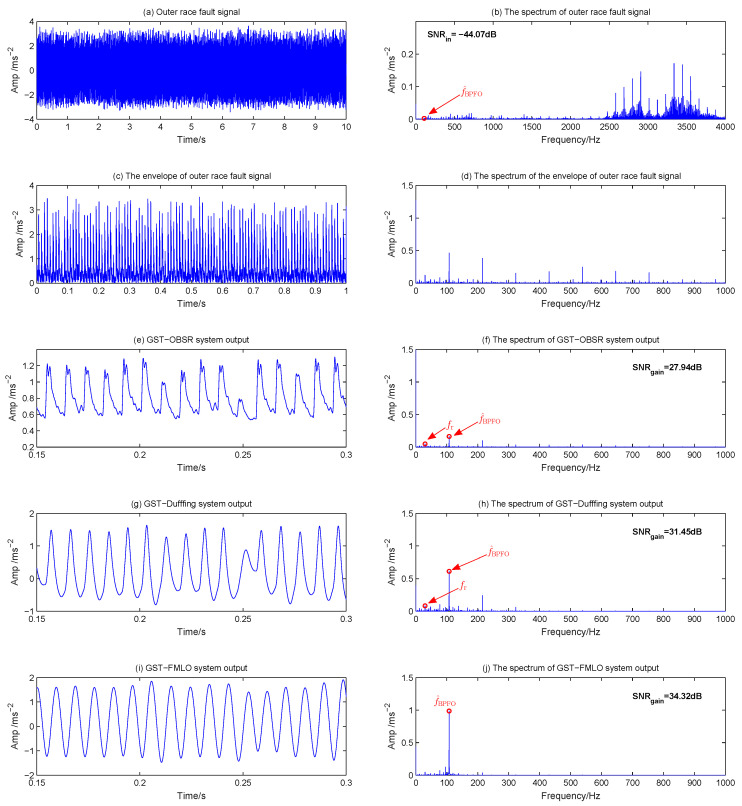
Outer race fault diagnosis by three different dynamical methods: (**a**) Outer race fault signal with ${\hat{f}}_{\mathrm{BPFO}}=107.6$ Hz; (**b**) The spectrum of outer race fault signal; (**c**) The envelope of outer race fault signal; (**d**) The spectrum of envelope signal; (**e**) GST-OBSR system output; (**f**) The spectrum of GST-OBSR system output; (**g**) GST-Duffing system output; (**h**) The spectrum of GST-Duffing system output; (**i**) GST-FMLO system output; (**j**) The spectrum of GST-FMLO system output.

In the fault diagnosis of outer race, the original waveform and spectrum of fault signal are described in Figure 14a,b, where the noise interference mainly concentrates in the bandwidth of 2400 to 3800 Hz, and the input SNR is calculated as −44.07 dB. When the envelope signal is analyzed in Figure 14c,d, it is seen that there still exist many harmonic interferences around the outer race fault frequency 107.6 Hz in the spectrum. Then, the envelope signal is used to drive the above three systems, and the time-domain outputs and the corresponding amplitude spectrums are shown in Figure 14e–j. It is found that the GST-FMLO system performs better to remove the low harmonics, and the component at ${\hat{f}}_{\mathrm{BPFO}}=107.6$ Hz is amplified the most. The SNR gain is 6.38 dB and 2.87 dB higher than that of GST-OBSR and GST-Duffing systems, respectively. This is also reflected in the optimal output waveform of GST-FMLO system. It behaves with good periodicity and and stationary amplitude. We can clearly identify the fault characteristic of outer race signal.

**Figure 15:**
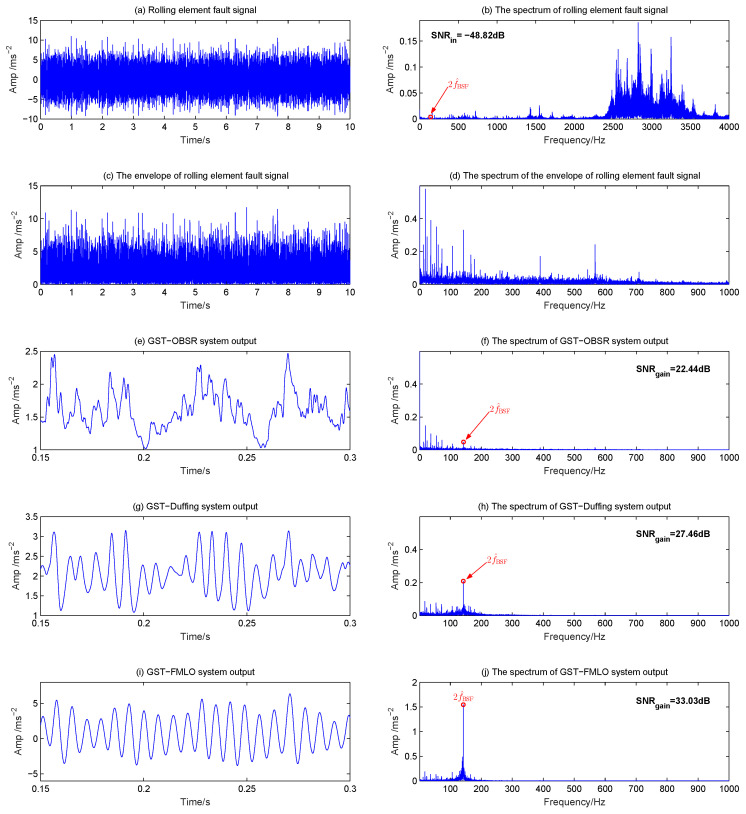
Rolling element fault diagnosis by three different dynamical methods: (**a**) Rolling element fault signal with $2{\hat{f}}_{\mathrm{BSF}}=141.8$ Hz; (**b**) The spectrum of rolling element fault signal; (**c**) The envelope of rolling element fault signal; (**d**) The spectrum of envelope signal; (**e**) GST-OBSR system output; (**f**) The spectrum of GST-OBSR system output; (**g**) GST-Duffing system output; (**h**) The spectrum of GST-Duffing system output; (**i**) GST-FMLO system output; (**j**) The spectrum of GST-FMLO system output.

In the diagnosis of rolling element, the fault signal is always surrounded by modulation sidebands at cage speed, and it is undoubtedly the most difficult to diagnose [42]. Here the original waveform and spectrum of fault signal are depicted in Figure 15a,b, where a large number of low harmonic interferences (in addition to the component at $f_{r}$) play the dominate role. The input SNR is as low as −48.82 dB, which causes the fault characteristic at $2{\hat{f}}_{\mathrm{BSF}}=141.8$ Hz to be much more difficult to identify. The results of envelope analysis and GST-OBSR system are shown in Figure 15c–f, respectively. It is clearly observed that many low harmonics are more obvious than characteristic harmonics, thus they are invalid. Although GST-Duffing system performs better and SNR gain is improved to 27.46 dB, as shown in Figure 15g,h, the interference is still relative heavy in the lower frequency band. Thus, the GST-FMLO system is further applied to diagnose the rolling element fault signal, in Figure 15i,j. We evidently observe the great improvement of output SNR, which results from the fact that, based on the analysis of multi-parameter regulation mechanism in Section 3.2, the system could play a cooperative role in optimally transforming the energy of SDN fluctuation to the fault signal. Thus, the component at 141.8 Hz significantly increases, and SNR gains are 10.59 dB and 5.57 dB higher than the GST-OBSR and GST-Duffing systems, respectively. It is fully demonstrated that the proposed GST-FMLO system in this paper is also an extremely effective method in the diagnosis of the rolling element.

It is noted that all the parameters in three systems are optimized by the adaptive PSO algorithm, and the results have been clearly listed in Table 2. Compared with the other two dynamical methods, the GST-FMLO system appears to be best in adaptively identifying the actual fault frequency and obtain the results with optimal performance.

Besides, a point worth emphasizing is that, although the regulation in GST-FMLO system involves more parameters, as a linear system it still seems to be most efficient, and thus we observe the shortest runtime, only about 16.7% of that in two other systems. In Table 2, all the values of $T_{\mathrm{sim}}$ are normalized by the runtime of GST-FMLO system. Obviously, the diagnosis efficiency is greatly increased by more than 5 times. The advantage mainly comes from the reduced algorithm complexity of linear system implementation. Thus, it provides an effective and efficient method in the practical applications, especially the online fault diagnosis.

## 5. Conclusions

This paper proposes an adaptive GST-FMLO system for bearing fault diagnosis in the framework of Langevin dynamics, which effectively extends the traditional nonlinear dynamical methods. By analyzing the system stationary response, in theory we discuss systematically the dependence of OAA *G* on various parameters, including damping coefficient γ, inherent frequency $\omega^{2}$, multiplicative SDN intensity $\sigma^{2}$, correlation rate λ, and GST coefficient *R*. It is found that the synergy of linear system, internal regulation and external driving can conduct a rich variety of non-monotonic behaviors, such as double-peak bona fide SR of G(R), single-peak GSR of G(γ), double-peak GSR of $G(\omega^{2})$, conventional SR of $G(\sigma^{2})$, and SI of G(λ). All these behaviors have a significant effect on optimizing the system parameters to improve the diagnosis performance of weak high-frequency signal in the heavy-noise background, and they are verified in the investigation of multi-parameter regulation mechanisms. Finally, three types of dynamical methods, i.e., GST-OBSR, GST-Duffing, and GST-FMLO systems, are applied to the experimental data, and the results show that the proposed GST-FMLO system has the best identifying ability, diagnosis performance and operating efficiency in all the fault diagnoses of inner race, outer race and rolling element. It is demonstrated that the method proposed in this paper has great potential in engineering applications.

## Figures and Tables

**Table 1:** The results of weak signal detection based on three different dynamical methods.

Method	Optimal System Parameters (osp)					Detection Performance		
	**osp-1**	**osp-2**	**osp-3**	**osp-4**	R	${\mathrm{SNR}}_{\mathrm{in}}$	${\mathrm{SNR}}_{\mathrm{out}}$	${\mathrm{SNR}}_{\mathrm{gain}}$
GST-OBSR	0.4680	0.0025	0.8440	-	$4.1561\times 10^{2}$	−34.10 dB	−24.48 dB	9.62 dB
GST-Duffing	0.2224	1.2144	0.2384	-	$4.4083\times 10^{2}$	−34.10 dB	−19.10 dB	15.00 dB
GST-FMLO	0.0407	1.5839	0.0812	0.7088	$3.9694\times 10^{2}$	−34.10 dB	−10.91 dB	23.19 dB

**Table 2:** The summaries of bearing fault diagnosis based on three different dynamical systems.

Method	Optimal System Parameters (osp)					Diagnosis Performance			
Inner race fault with ${\hat{f}}_{\mathrm{BPFI}}=161.9$ Hz									
	osp-1	osp-2	osp-3	osp-4	*R*	${\mathrm{SNR}}_{\mathrm{in}}$	${\mathrm{SNR}}_{\mathrm{out}}$	${\mathrm{SNR}}_{\mathrm{gain}}$	$T_{\mathrm{sim}}$
GST-OBSR	1.7930	0.0036	0.8986	-	$1.1411\times 10^{3}$	−44.46 dB	−19.55 dB	24.91 dB	6.60
GST-Duffing	0.2345	1.8715	0.5370	-	$5.0625\times 10^{2}$	−44.46 dB	−14.44 dB	30.02 dB	6.74
GST-FMLO	0.0205	1.7558	0.0272	0.6580	$5.8626\times 10^{2}$	−44.46 dB	−9.85 dB	34.61 dB	1.00
Outer race fault with ${\hat{f}}_{\mathrm{BPFO}}=107.6$ Hz									
	osp-1	osp-2	osp-3	osp-4	*R*	${\mathrm{SNR}}_{\mathrm{in}}$	${\mathrm{SNR}}_{\mathrm{out}}$	${\mathrm{SNR}}_{\mathrm{gain}}$	$T_{\mathrm{sim}}$
GST-OBSR	1.6615	0.0096	1.0263	-	$8.4209\times 10^{2}$	−44.07 dB	−16.14 dB	27.94 dB	6.58
GST-Duffing	0.5250	0.0878	1.4789	-	$6.1283\times 10^{2}$	−44.07 dB	−12.62 dB	31.45 dB	6.72
GST-FMLO	0.1196	1.7923	0.0071	0.9982	$3.7154\times 10^{2}$	−44.07 dB	−9.76 dB	34.32 dB	1.00
Rolling element fault with $2{\hat{f}}_{\mathrm{BSF}}=141.8$ Hz									
	osp-1	osp-2	osp-3	osp-4	*R*	${\mathrm{SNR}}_{\mathrm{in}}$	${\mathrm{SNR}}_{\mathrm{out}}$	${\mathrm{SNR}}_{\mathrm{gain}}$	$T_{\mathrm{sim}}$
GST-OBSR	1.6864	0.0537	0.5879	-	$3.6795\times 10^{2}$	−48.82 dB	−26.38 dB	22.44 dB	6.51
GST-Duffing	0.4721	1.7500	0.6105	-	$3.7652\times 10^{2}$	−48.82 dB	−21.36 dB	27.46 dB	7.02
GST-FMLO	0.0817	1.7770	0.0305	0.8001	$4.8979\times 10^{2}$	−48.82 dB	−15.79 dB	33.03 dB	1.00
